# The link between vitamin D, chemerin and metabolic profile in overweight and obese children - preliminary results

**DOI:** 10.3389/fendo.2023.1143755

**Published:** 2023-04-20

**Authors:** Maria Krajewska, Ewelina Witkowska-Sędek, Małgorzata Rumińska, Anna M. Kucharska, Anna Stelmaszczyk-Emmel, Maria Sobol, Anna Majcher, Beata Pyrżak

**Affiliations:** ^1^ Department of Paediatrics and Endocrinology, Medical University of Warsaw, Warsaw, Poland; ^2^ Department of Laboratory Diagnostics and Clinical Immunology of Developmental Age, Medical University of Warsaw, Warsaw, Poland; ^3^ Department of Biophysics, Physiology and Pathophysiology, Medical University of Warsaw, Warsaw, Poland

**Keywords:** obesity, children, vitamin D, chemerin, C-reactive protein, glucose, insulin, lipid profile

## Abstract

**Background:**

Vitamin D affects adipogenesis, oxidative stress, inflammation, secretion of adipocytokines, lipid metabolism and thermogenesis. Some researchers postulate that those effects could be exerted by the influence of vitamin D on chemerin levels.

**Aim of the study:**

We aimed to investigate if there is a link between serum 25-hydroksyvitamin D [25(OH)D], chemerin and metabolic profile in overweight and obese children before and after vitamin D supplementation.

**Material and methods:**

The prospective study included 65 overweight and obese children aged 9.08-17.5 years and 26 peers as a control. None of the patients in the study group had received vitamin D within the last twelve months before the study.

**Results:**

The study group had lower baseline 25(OH)D (p<0.001) and higher chemerin (p<0.001), triglycerides (TG, p<0.001), triglycerides/high density lipoprotein cholesterol (TG/HDL-C, p<0.001), C-reactive protein (CRP, p<0.05), fasting insulin (p<0.001), Homeostasis Model Assessment - Insulin Resistance (HOMA-IR, p<0.001), alanine aminotransferase (ALT, p<0.001) and uric acid (p<0.001) compared to the control group. Baseline vitamin D was related to fasting insulin (R=-0.29, p=0.021), HOMA-IR (R=-0.30, p=0.016), HDL-C (R=0.29, p=0.020) and uric acid (R=-0.28, p=0.037) in the study group. Baseline chemerin was related to insulin at 30’ (R=0.27, p=0.030), 60’ (R=0.27, p=0.033), 90’ (R=0.26, p=0.037) and 120’ (R=0.26, p=0.040) during the oral glucose tolerance test (OGTT) and ALT (R=0.25, p=0.041) in the study group. Correlation between vitamin D and chemerin (R=-0.39, p=0.046) was found only in the control group. After six months of vitamin D supplementation a decrease in CRP (p<0.01), total cholesterol (p<0.05), ALT (p<0.01), glucose at 150’ OGTT (p<0.05) was observed. Moreover, we noticed a tendency for negative association between 25(OH)D and chemerin levels (p=0.085). Multivariable backward linear regression models were build using baseline vitamin D, baseline chemerin and six months chemerin as the dependent variables.

**Conclusions:**

Our study confirmed that vitamin D has positive effect on metabolic profile in overweight and obese children. The relationship between vitamin D and chemerin is not clear, nevertheless we have observed a tendency to decrease chemerin concentrations after improving vitamin D status, even without a significant reduction in body fat mass.

## Introduction

Adipose tissue as a highly metabolically active organ has been intensively studied over the last decades (1, 2). It secretes a number of adipokines and pro-inflammatory cytokines which link excess body fat with chronic inflammation and atherosclerosis, contributing to the development of obesity-related metabolic disorders (1, 3–5). Vitamin D is also involved in those processes (6, 7). The development of obesity and vitamin D status are mutually dependent (6). It has been confirmed that vitamin D can affect both genomic and nongenomic responses in adipose tissue. Its effect includes the impact on adipogenesis and apoptosis, on the development of oxidative stress and inflammation, the regulation of the secretion of adipokines, pro-inflammatory and anti-inflammatory cytokines, the influence on lipid metabolism and thermogenesis (8). Some studies reported the beneficial effect of vitamin D supplementation on reducing cardiometabolic risk factors in childhood obesity (9–11). Some researchers postulated that the protective effect of vitamin D on metabolic profile could be exerted by the influence on chemerin levels (12–14). Chemerin is one of the most important multifunctional adipokines, generated mainly in subcutaneous and visceral adipose tissue by elastase and tryptase which activate prochemerin. Chemerin mRNA is expressed also in fibroblasts, chondrocytes, epithelial cells, platelets and in a number of organs such as liver, female reproductive organs, adrenal glands, lungs, kidneys and pancreas (15–18). Chemerin is involved in glucose homeostasis, lipid metabolism, maintenance of energy balance, adipogenesis, angiogenesis, inflammatory and autoimmune processes (1, 18–22). Recent studies indicate that it could be also used as a marker of tumours (23, 24). Increased chemerin levels, typical for obesity, are associated with adiposity-related dyslipidaemia, insulin resistance, low-grade inflammation and hypertension (25–29). Recent evidence suggest that chemerin, similarly to other adipokines e.g. leptin, may influence bone metabolism. Experimental data indicate that chemerin promotes osteoclastogenesis (30–35).

The aim of these study was to investigate the link between serum 25-hydroksyvitamin D [25(OH)D], chemerin and metabolic profile in overweight and obese children before and after vitamin D supplementation.

## Material and methods

The prospective study included 65 children (51 obese and 14 overweight, aged 9.08-17.5 years) with mean body mass index (BMI) 30.9 ± 4.8 and 26 peers as a control with mean BMI 18.3 ± 2.6 age- and sex- matched. None of the patients in the study group had received vitamin D within the last twelve months before the study. The study protocol was approved by the Bioethics Committee at the Medical University of Warsaw (decision number KB/257/2013) and conducted in the Department of Paediatrics and Endocrinology at the Medical University of Warsaw (Poland). At the time of blood collection, children in both the study and the control group were healthy, without infection or chronic diseases and were not taking any medication. During the study period the participants did not change their diet or the level of physical activity. Serum 25(OH)D, chemerin, C-reactive protein (CRP), glucose and insulin during the oral glucose tolerance test (OGTT), uric acid, aminotransferases (aspartate aminotransferase – AST and alanine aminotransferase - ALT), lipid profile (total cholesterol, low density lipoprotein cholesterol - LDL-C, high density lipoprotein cholesterol - HDL-C, triglycerides - TG) and glycated haemoglobin (HbA1c) were determined at baseline (both in the study and in the control group) and after six months of vitamin D supplementation (in the study group). Following indices were calculated: TG/HDL-C ratio, Homeostasis Model Assessment - Insulin Resistance (HOMA-IR) and Quantitative Insulin Sensitivity Check Index (QUICKI) at baseline and after six months of vitamin D supplementation. The aim of vitamin D administration was to achieve the reference serum 25(OH)D levels between 30 and 50 ng/ml after six months of intervention (36). Depending on the serum 25(OH)D levels, the doses of vitamin D ranged from 2000 to 4000 units per day. Serum 25(OH)D concentrations were assessed every month, which allowed us to control compliance and to modify administered vitamin D doses to achieve reference values after six months of the study.

Anthropometric parameters (height, weight, waist and hip circumference) were measured using standardized methods. Based on these measurements BMI, waist-to-hip ratio (WHR) and waist-to-height ratio (WHtR) were calculated. The skinfold thickness (mm) was measured under the triceps brachii muscle and under the inferior scapular angle. Body fat percentage (%FAT skinfolds) was calculated in the study group and in the control group using Slaughter formula (37). Additionally, in the study group the percentage of fat was measured using a bioimpedance analysis (%FAT BIA) device (Maltron Body FAT Analyzer BF-905). Height and weight were evaluated according to polish 2010 growth references for school-aged children and adolescents (38). The degree of obesity expressed as BMI standard deviation score (SDS) was calculated using the LMS method to normalize skewness of the distribution of BMI (38, 39). Obesity was defined as BMI SDS ≥ 2, and overweight as BMI SDS ≥ 1 and < 2 (40).

### Biochemical analyses

Blood samples were collected after overnight fasting and analysed by standard methods. Serum 25(OH)D levels (ng/ml) were determined by immunoassay method using Architect Analyzer (Abbott Diagnostics, Lake Forest, USA). Serum levels of chemerin (pg/ml) were evaluated by ELISA (Mediagnost, Reutlingen, Germany) using Asys UVM 340 Analyzer. The concentrations of fasting glucose (mg/dl) and glucose in the oral glucose tolerance test (OGTT 1.75g of glucose/kg body weight, no more than 75g; blood samples taken at 0’, 30’, 60’, 90’, 120’, 150’, 180’) were determined in blood serum by glucose oxidase colorimetric method using Vitros 5600 Analyzer (Ortho Clinical Diagnostic, New Jersey, USA). The concentrations of HbA1c (%) were measured in whole blood by ion-exchange high-performance liquid chromatography using D-10 Hemoglobin Analyzer (BIO-RAD). The concentrations of insulin (uIU/ml) were measured in serum by immunoassay using IMMULITE 2000 Xpi Analyzer (Siemens). Serum levels of total cholesterol (mg/dl), LDL-C (mg/dl), HDL-C (mg/dl), TG (mg/dl), ALT (U/l), AST (U/l), CRP (mg/dl) and uric acid (mg/dl) were determined in blood serum using Vitros 5600 Analyzer (Ortho Clinical Diagnostic, New Jersey, USA). The HOMA-IR was calculated as follows: HOMA-IR = (fasting glucose mg/dl) x (fasting insulin uIU/ml)/405. The QUICKI was calculated as follows: QUICKI = 1/[log(fasting insulin uIU/ml) + log(fasting glucose mg/dl)] (41). The levels of uric acid were classified as normal or increased according to the reference values provided by the manufacturer. The values of lipid parameters were classified as normal or increased/decreased (increased total cholesterol, LDL-C, TG and decreased HDL-C) according to the reference values used in paediatric population (42).

### Statistical analysis

Statistical analysis was performed using Statistica 13.3. Data distribution was checked using the Shapiro–Wilk test. Data were presented as means with standard deviation or the median and interquartile ranges, as appropriate. Comparisons between baseline data of the study group and the control group were made using the T-test for parametric data or using the U Mann-Whitney test for non-parametric data. Analysis of changes of the same parameter at baseline and after six months of vitamin D supplementation were provided using the T-test or the Wilcoxon test, as appropriate. Correlation analysis was performed using Spearman correlation coefficient. In further analysis, we used multivariable stepwise regression analysis to determine which factors (model first: body mass SDS, hip circumference, BMI SDS, waist circumference or model second: fasting insulin, HOMA-IR, HDL-C, uric acid) were associated with baseline 25(OH)D (as dependent variable). We also analysed which parameters (model third: %FAT BIA, %FAT skinfolds, WHtR, BMI SDS or model fourth: insulin at 60’ and 120’ during the OGTT, ALT, TG/HDL-C) were associated with baseline chemerin levels (as dependent variable). In model fifth: WHR, WHtR, %FAT skinfolds and model sixth: TG, TG/HDL-C, ALT we investigated which factors were associated with chemerin values after six months of vitamin D supplementation.

## Results

### Baseline anthropometric and biochemical parameters in the study group and in the control group

Baseline anthropometric and biochemical characteristics of the study group and of the control group are presented in **Tables 1**, **2**. As expected, baseline 25(OH)D levels were significantly lower in children with excess body weight compared to the control group (median values 16.0 vs. 25.7 ng/ml, p<0.001), whereas baseline chemerin levels were significantly higher in the study group than in the control group (median 212.0 vs. 147.1 pg/ml, p<0.001). We noticed also that the study group had higher TG (p<0.001), TG/HDL-C ratio (p<0.001), CRP (p<0.05), fasting insulin (p<0.001), HOMA-IR (p<0.001), ALT activity (p<0.001) and uric acid (p<0.001) compared to the control group. The levels of HDL-C were significantly lower (p<0.001) in overweight and obese children.

**Table 1:** Baseline anthropometric measurements in the study group and in the control group.

	STUDY GROUP (n = 65)	CONTROL GROUP (n 26)	p value
**Age (years)**	13.4 ± 2.11	13.5 ± 2.39	ns
**Height SDS**	0.6 ± 1.25	-0.7 ± 1.47	< 0.001
**Weight SDS**	2.3 ± 0.69	-0.4 ± 1.15	< 0.001
**WC (cm)**	90.9 ± 10.27	61.6 ± 6.53	< 0.001
**HC (cm)**	106.2 ± 10.60	77.1 ± 8.35	< 0.001
**WHR**	0.9 ± 0.06	0.8 ± 0.04	< 0.001
**WHtR**	0.56 (0.52-0.60)	0.41 (0.39-0.44)	< 0.001
**% FAT (skinfolds)**	35.7 (32.1-39.7)	23.1 (19.6-24.7)	< 0.001
**BMI SDS**	2.3 ± 0.47	-0.3 ± 0.83	< 0.001

Data are presented as means ± standard deviations score or as median with interquartile range, as appropriate. SDS, standard deviation score; WC, waist circumference; HC, hip circumference; WHR, waist-to-hip ratio; WHtR, waist-to-height ratio; % FAT (skinfolds), percentage of body fat estimated from skinfolds; BMI, body mass index; ns, not significant.

**Table 2:** Baseline biochemical parameters in the study group and in the control group.

	STUDY GROUP (n = 65)	CONTROL GROUP (n = 26)	p value
**25(OH)D (ng/ml)**	16.0 (12.6 - 20.0)	25.7 (18.6 - 31.1)	< 0.001
**Total cholesterol (mg/dl)**	161.0 (142.0 - 181.0)	160.5 (138.0 - 170.0)	ns
**Triglycerides (TG) (mg/dl)**	108.0 (83.0 - 161.0)	68.0 (53.0 - 93.0)	< 0.001
**TG/HDL-C**	2.5 (2.0 - 4.2)	1.1 (0.7 - 1.7)	< 0.001
**LDL-C (mg/dl)**	94.3 ± 27.1	84.2 ± 29.1	ns
**HDL-C (mg/dl)**	43.4 ± 11.1	60.3 ± 10.7	< 0.001
**CRP (mg/dl)**	0.5 (0.5 - 0.7)	0.5 (0.5 - 0.5)	< 0.01
**Glucose fasting (mg/dl)**	86.0 (82.0 - 91.0)	84.0 (78.0 - 86.0)	ns
**Insulin fasting (uIU/ml)**	14.7 (9.1 - 25.6)	7.2 (2.9 - 11.7)	< 0.001
**HOMA-IR**	3.0 (1.9 - 5.4)	1.5 (0.6 - 2.6)	< 0.001
**QUICKI**	0.32 (0.30 - 0.35)	0.36 (0.33 - 0.42)	< 0.001
**HbA1c (%)**	5.2 (5.0 - 5.35)	5.1 (4.9 - 5.3)	ns
**chemerin (pg/ml)**	212.0 (177.5 - 238.3)	147.1 (128.6 - 171.3)	< 0.001
**AST (U/I)**	28.0 (23.0 - 35.0)	25.0 (22.0 - 33.5)	ns
**ALT (U/I)**	27.0 (21.0 - 36.0)	18.5 (13.5 - 22.0)	< 0.001
**Uric acid (mg/dl)**	5.85 (5.3 - 6.8)	4.6 (3.9 - 5.2)	< 0.001

Data are presented as means ± standard deviations score or as median with interquartile range, as appropriate. 25(OH)D, 25-hydroxyvitamin D; TG, triglycerides; LDL-C, low density lipoprotein cholesterol; HDL-C, high density lipoprotein cholesterol; CRP, C-reactive protein; HOMA-IR, Homeostasis Model Assessment- Insulin Resistance, QUICKI, Quantitative Insulin Sensitivity Check Index; HbA1c, Glycated Hemoglobin, AST, aspartate aminotransferase; ALT, alanine aminotransferase; ns, not significant.

Correlation analysis revealed that baseline 25(OH)D levels correlated significantly with nutritional status parameters of the study group such as body weight SDS (R=-0.27, p=0.032), BMI SDS (R=-0.27, p=0.028), hip circumference (R=-0.27, p=0.030). In the control group none associations between baseline vitamin D status and anthropometric parameters were observed.

**Figure 1:**
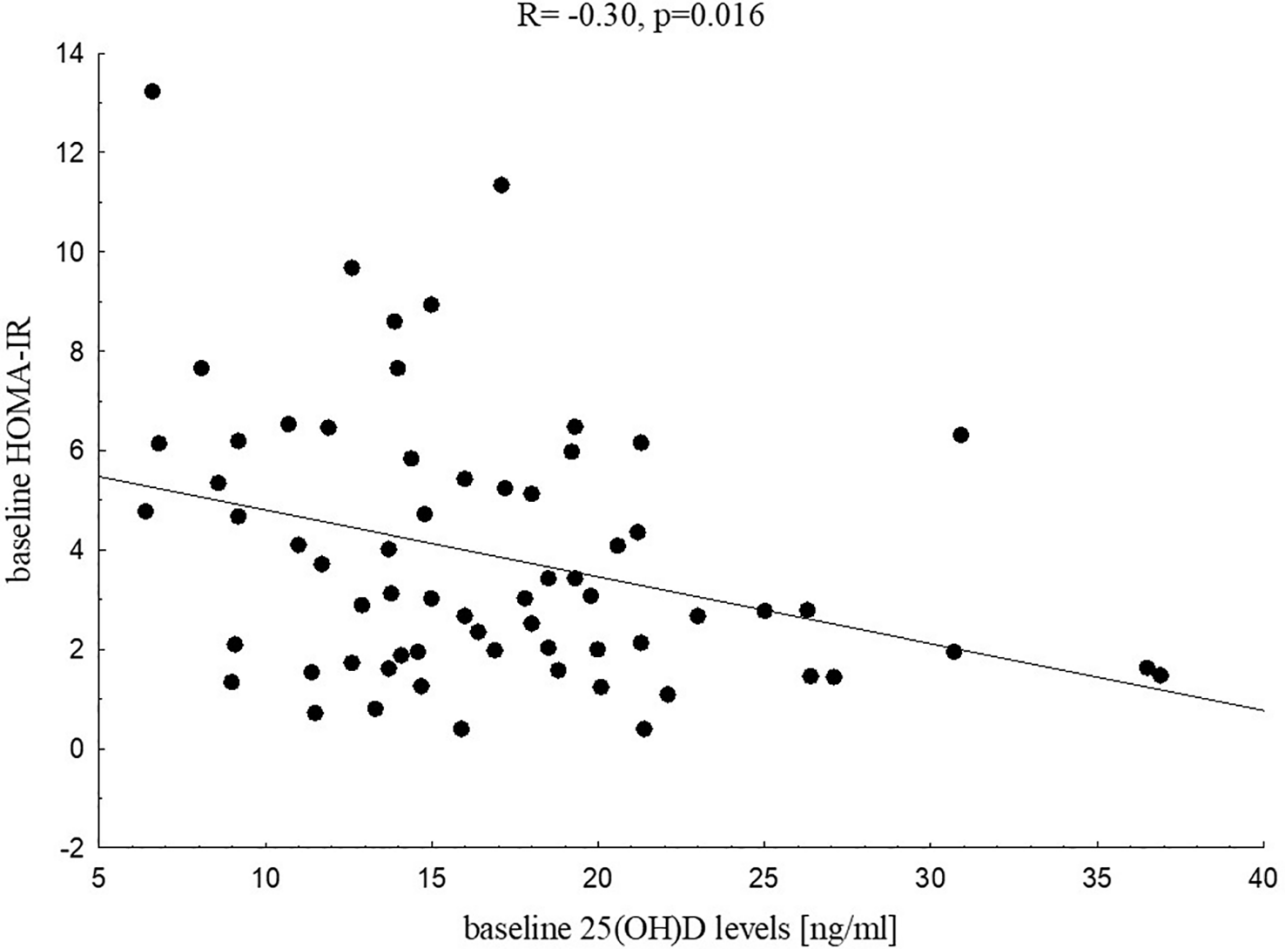
Correlation between 25(OH)D levels and HOMA-IR in the study group at baseline.

**Figure 2:**
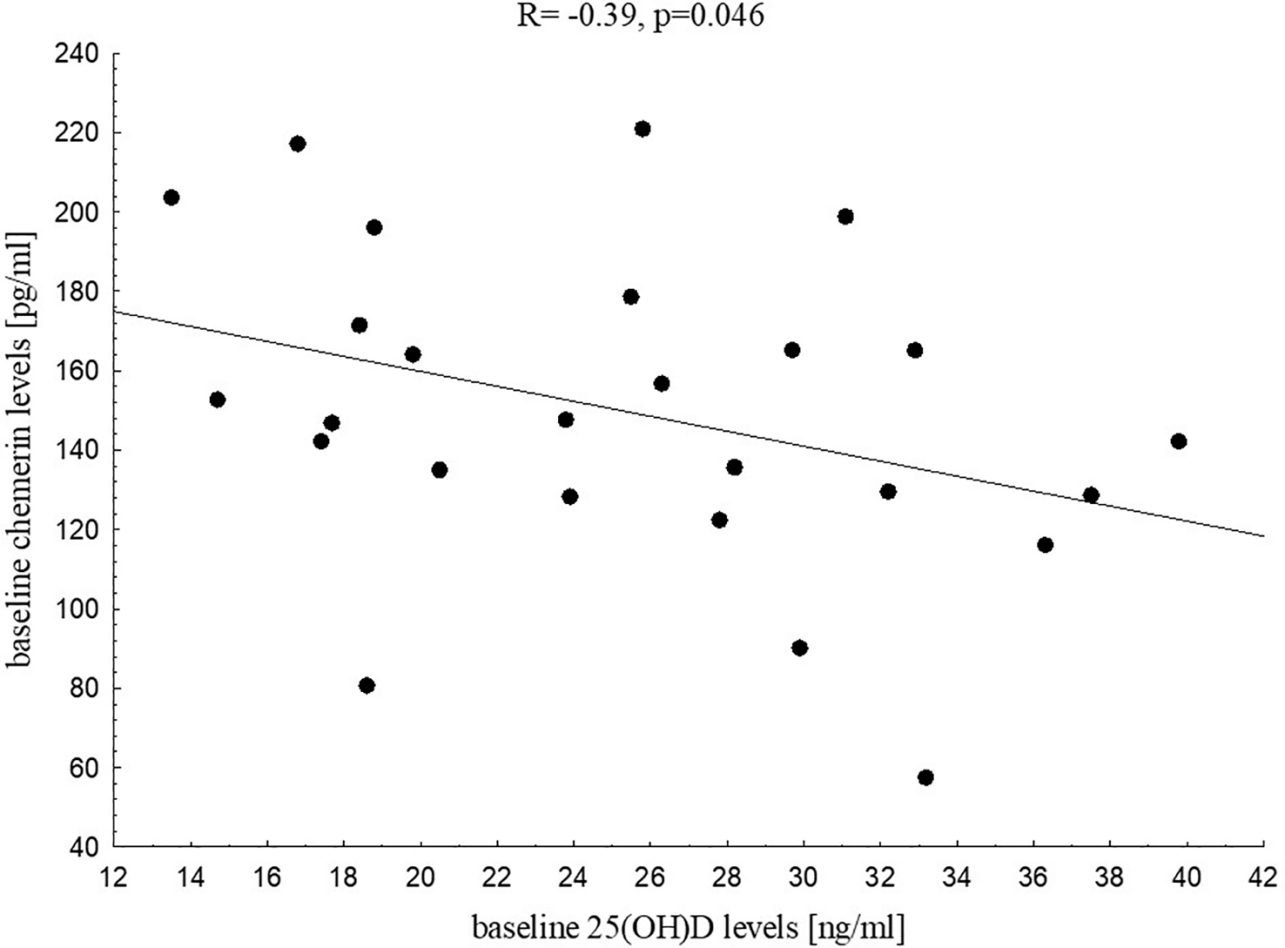
Correlation between 25(OH)D and chemerin levels in the control group at baseline.

Analysing baseline metabolic profile of both groups we found that vitamin D status was related significantly to fasting insulin (R=-0.29, p=0.021), HOMA-IR (R=-0.30, p=0.016, **Figure 1**), QUICKI (R=0.29, p=0.020), HDL-C (R=0.29, p=0.020) and uric acid (R=-0.28, p=0.037) in the study group. These associations were not found in the control group. We did not find any associations between 25(OH)D levels and CRP, fasting glucose or glucose levels in OGTT, HbA1c, aminotransferase activity both in the study group and the control group at baseline. Interestingly, we noticed, but only in the control group, significant negative correlation between baseline vitamin D level and baseline chemerin (R=-0.39, p=0.046, **Figure 2**).

Baseline chemerin levels were related to WHtR (R=0.32, p=0.010), %FAT BIA (R=0.25, p=0.045), %FAT skinfolds (R=0.26, p=0.042), insulin at 30’ (R=0.27, p=0.030), 60’ (R=0.27, p=0.033), 90’ (R=0.26, p=0.037) and 120’ during the OGTT (R=0.26, p=0.040) and ALT activity (R=0.25, p=0.041) in the study group. In the control group we found only positive relationship between baseline chemerin level and WHR (R=0.46, p=0.045).

### Anthropometric and biochemical parameters after six months of vitamin D supplementation in the study group

Comparison between anthropometric and biochemical parameters in the study group at baseline and after six months of vitamin D supplementation is presented in **Tables 3**, **4**. After six months of vitamin D supplementation the levels of 25(OH)D significantly increased in the study group compared to baseline values (median 16.0 vs. 27.1 ng/ml, p<0.001), while BMI SDS and chemerin levels did not change significantly. We found significant decrease in CRP (p<0.01), total cholesterol (p<0.05), ALT (p<0.01) and AST (p<0.01). We also noticed a decrease in glucose at 150’ during the OGTT (p<0.05) and a tendency for lower glucose levels at 120’ during the OGTT (p=0.079).

**Table 3:** Comparison between anthropometric parameters in the study group at baseline and after six months of vitamin D supplementation.

	BASELINE	SIX MONTHS	p value
**Height SDS**	0.6 ± 1.25	0.6 ± 1.25	ns
**Weight SDS**	2.3 ± 0.67	2.2 ± 0.76	ns
**WC (cm)**	90.9 ± 10.27	89.7 ± 11.07	ns
**HC (cm)**	106.2 ± 10.60	106.6 ± 11.80	ns
**WHR**	0.9 ± 0.06	0.9 ± 0.05	ns
**WHtR**	0.56 (0.5-0.6)	0.54 (0.5-0.6)	< 0.001
**% FAT (skinfolds)**	35.7 (32.1-39.7)	34.3 (29.9-38.1)	< 0.001
**% FAT BIA**	40.4 ± 7.61	38.3 ± 8.93	ns
**BMI SDS**	2.3 ± 0.47	2.2 ± 0.56	ns

Data are presented as means ± standard deviations score or as median with interquartile range, as appropriate. SDS, standard deviation score; WC, waist circumference; HC, hip circumference; WHR, waist-to-hip ratio; WHtR, waist-to-height ratio; %FAT (skinfolds), percentage of body fat estimated from skinfolds; %FAT BIA, percentage of body fat estimated using bioelectrical impedance analysis method; BMI, body mass index; ns, not significant.

**Table 4:** Comparison between biochemical parameters in the study group at baseline and after six months of vitamin D supplementation.

	BASELINE	SIX MONTHS	p value
**25(OH)D (ng/ml)**	16.0 (12.6 - 20.0)	27.1 (22.9 - 32.3)	< 0.001
**Total cholesterol (mg/dl)**	161.0 (142.0 - 181.0)	156.0 (139.0 - 171.0)	<0.05
**Triglycerides (TG) (mg/dl)**	108.0 (83.0 - 161.0)	112.0 (84.0 - 150.0)	ns
**TG/HDL-C ratio**	2.5 (2.0-4.2)	2.7 (1.9 - 3.7)	ns
**LDL-C (mg/dl)**	94.3 ± 27.1	88.8 ± 25.8	ns
**HDL-C (mg/dl)**	43.4 ± 11.1	44.3 ± 11.8	ns
**CRP (mg/dl)**	0.5 − (0.5-0.7)	0.5 (0.5 - 0.5),	<0.01
**Glucose fasting (mg/dl)**	86.0 (82.0 - 91.0)	85.2 (82.0 - 88.3)	ns
**Glucose 30' OGTT (mg/dl)**	139.0 (127.0 - 156.3)	135.5 (122.2 - 155.8)	ns
**Glucose 60' OGTT (mg/dl)**	120.0 (106.0 - 150.5)	120.6 (103.7 - 141.4)	ns
**Glucose 90' OGTT (mg/dl)**	116.0 (102.9 - 136.8)	112.0 (99.0 - 127.0)	ns
**Glucose 120' OGTT (mg/dl)**	110.0 (99.4 - 125.4)	105.8 (92.6 - 120.0)	ns
**Glucose 150' OGTT (mg/dl)**	91.0 (79.0 - 106.2)	85.4 (76.0 - 104.0)	<0.05
**Glucose 180' OGTT (mg/dl)**	75.9 (67.5 - 93.5)	75.0 (67.0 - 85.0)	ns
**Insulin fasting (uIU/ml)**	14.7 (9.1 - 25.6)	14.6 (9.9 - 23.6)	ns
**Insulin 30' OGTT (uIU/ml)**	93.7 (66.1 - 152.0)	91.8 (70.4 - 178.0)	ns
**Insulin 60' OGTT (uIU/ml)**	92.2 (62.8 - 143.0)	96.9 (67.8 - 145.0)	ns
**Insulin 90' OGTT (uIU/ml)**	89.8 (58.3 - 135.0)	81.7 (53.8 - 124.0)	ns
**Insulin 120' OGTT (uIU/ml)**	76.3 (54.6 - 121.0)	72.0 (49.5 - 122.0)	ns
**Insulin 150' OGTT (uIU/ml)**	61.3 (34.3 - 108.0)	45.9 (33.1 - 85.8)	ns
**Insulin 180' OGTT (uIU/ml)**	35.0 (20.3 - 56.3)	25.3 (17.6 - 45.3)	ns
**HOMA-IR**	3.0 (1.9 - 5.4)	3.4 (2.0 - 5.0)	ns
**QUICKI**	0.3 (0.3 - 0.4)	0.3 (0.3 - 0.3)	ns
**HbAle (%)**	5.2 (5.0 - 5.4)	5.2 (5.0 - 5.5)	ns
**chemerin (pg/ml)**	212.0 (177.5 - 238.3)	206.8 (180.5 - 236.0)	ns
**AST (U/I)**	28.0 (23.0 - 35.0)	25.0 (23.0 - 30.0)	<0.01
**ALT (U/I)**	27.0 (21.0 - 36.0)	25.5 (20.0 - 35.0)	< 0.01
**Uric acid (mg/dl)**	5.85 (5.3 - 6.8)	5.9 (5.3 - 6.9)	ns

Data are presented as means ± standard deviations score or as median with interquartile range, as appropriate. 25(OH)D, 25-hydroxyvitamin D; TG, triglycerides; LDL-C, low density lipoprotein cholesterol; HDL-C, high density lipoprotein cholesterol; CRP, C-reactive protein; HOMA-IR, Homeostasis Model Assessment- Insulin Resistance, QUICKI, Quantitative Insulin Sensitivity Check Index; HbA1c, Glycated Hemoglobin, AST, aspartate aminotransferase; ALT, alanine aminotransferase; ns, not significant.

Although we did not find any relationship between vitamin D status after six months of its supplementation and anthropometric or biochemical parameters at six months, we noticed a tendency for negative association between 25(OH)D and chemerin levels (p=0.085).

**Figure 3:**
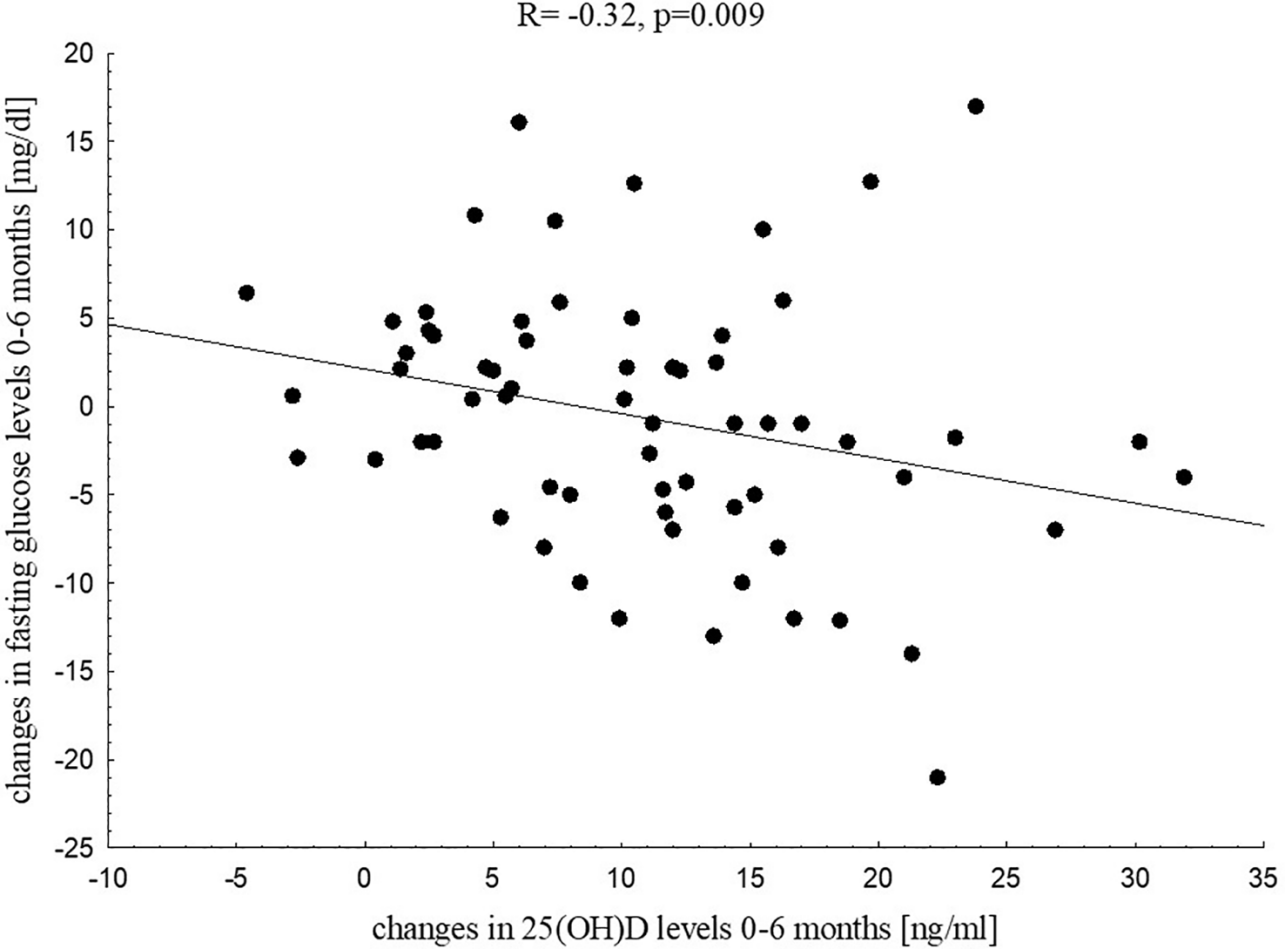
Correlation between changes in 25(OH)D levels and changes in fasting glucose in the study group after six months of vitamin D supplementation.

In further analysis we aimed to investigate if changes in vitamin D status during its supplementation influence metabolic profile in children with excess body mass. We found that changes in 25(OH)D levels were related negatively to changes in fasting glucose (R=-0.32, p=0.009, **Figure 3**) and changes in insulin levels at 120’ during the OGTT (R=-0.26, p=0.036).

**Figure 4:**
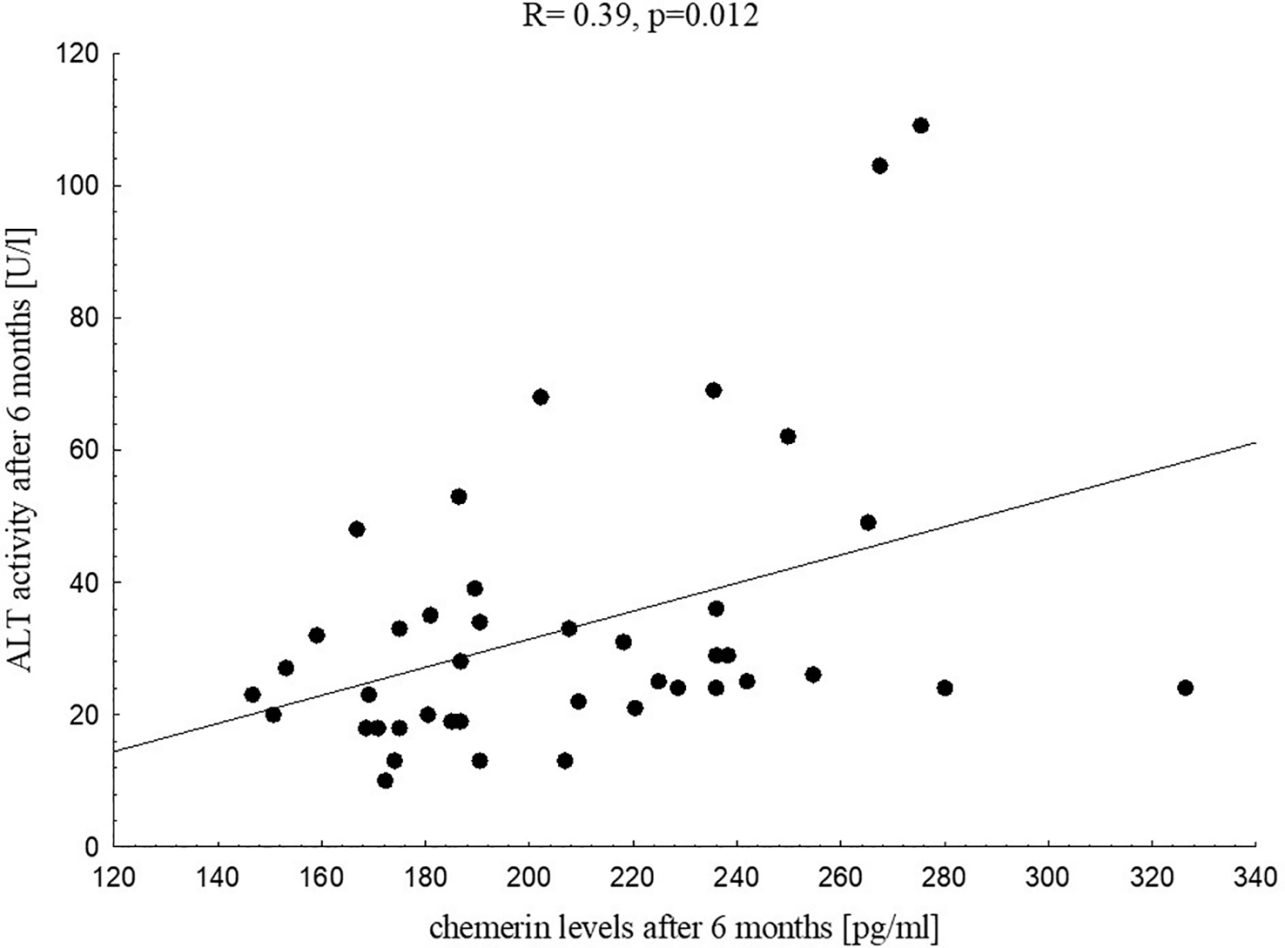
Correlation between chemerin levels and ALT activity in the study group after six months of vitamin D supplementation.

After six months of vitamin D supplementation chemerin levels correlated significantly with WHR (R=0.42, p=0.002), WHtR (R=0.38, p=0.006) and tended to be positively associated with % FAT skinfolds (p=0.052) and waist circumference (p=0.068). Among biochemical parameters six months chemerin levels correlated significantly with insulin at 30’ during the OGTT (R=0.25, p=0.043), TG (R=0.25, p=0.042), TG/HDL-C ratio (R=0.25, p=0.044) and ALT (R=0.39, p=0.012, **Figure 4**) in that period.

We investigated also how many patients in the study group presented dyslipidaemia and hyperuricemia, and if there were any significant associations between vitamin D and chemerin in these subgroups. At baseline dyslipidaemia was observed in 87% of children and hyperuricemia in 37,5%. After six months of vitamin D supplementation dyslipidaemia was found in 83% of the study group and hyperuricemia in 30%. Unfortunately, we did not find any statistically significant relationships.

### Summary of the multivariable backward linear regression models build for the study group

Based on findings of Spearman correlation analysis we build some multivariable backward linear regression models for the study group using baseline 25(OH)D values, baseline chemerin and six months chemerin values as the dependent variables, as appropriate.

The first model included baseline vitamin D (as dependent variable) and baseline anthropometric parameters such as body mass SDS, hip circumference, BMI SDS and waist circumference as independent variables. This model was statistically significant with cumulative R^2 ^= 0.10, p=0.011 and identified hip circumference as the parameter significantly negatively associated with baseline 25(OH)D levels. The received correlation coefficient was equal β=-0.320 ± 0.122 with 95% CI from -0.564 to -0.075.

The second model included baseline vitamin D (as dependent variable) and baseline fasting insulin, HOMA-IR, HDL-C and uric acid as independent variables. The model was statistically significant with cumulative R^2^ = 0.20, p=0.001 and identified only HDL-C level as the parameter significantly positively associated with baseline 25(OH)D level. The received correlation coefficient was equal β=0.443 ± 0.122 with 95% CI from 0.199 to 0.688.

Next two models included baseline chemerin level as dependent variable and respectively baseline % FAT BIA, % FAT skinfolds, WHtR and BMI SDS (third model) and baseline insulin at 60’ during the OGTT, insulin at 120’ during the OGTT, ALT and TG/HDL-C as independent variables (fourth model). The third model was statistically significant with cumulative R^2^ = 0.09, p=0.023. In that model WHtR was identified as one factor significantly positively associated with baseline chemerin. The received correlation coefficient was equal β=0.294 ± 0.126 with 95% CI from 0.043 to 0.545. The fourth model wasn’t statistically significant with cumulative R^2^ = 0.03, p=0.814.

Chemerin values after six months of vitamin D supplementation were used as dependent variable in the fifth (WHR, WHtR, % FAT skinfolds at six months as independent variables) and sixth model (TG, TG/HDL-C, ALT at six months as independent variables). The fifth model was statistically significant with cumulative R^2^ = 0.14, p=0.007 and identified WHtR at six months of vitamin D supplementation as a parameter significantly positively associated with chemerin level in that period. The received correlation coefficient was equal β=0.379 ± 0.135 with 95% CI from 0.108 to 0.651. The sixth model was statistically significant with cumulative R^2^ = 0.14, p=0.016 and revealed ALT as a factor significantly positively associated with chemerin at six months. The received correlation coefficient was equal β=0.378 ± 0.150 with 95% CI from 0.073 to 0.682.

## Discussion

In our study we aimed to investigate the relationships between vitamin D status, chemerin and metabolic prolife parameters among overweight and obese children. Our analysis confirmed significantly lower vitamin D levels coincided with higher chemerin levels in patients with excess body fat compared to the controls, but we did not find any direct relationships between vitamin D status and chemerin in those children. This results stand in line with previous studies (25, 43–46). We noticed that baseline vitamin D was related mainly to fasting insulin, insulin resistance indices, HDL-C and uric acid, whereas baseline chemerin was positively associated with insulin secretion after glucose intake in OGTT and ALT activity in the study group. Both vitamin D and chemerin were related to nutritional status parameters, vitamin D mainly to BMI SDS and hip circumference, while chemerin was related to WHtR and fat mass expressed as %FAT BIA and %FAT skinfolds. Interestingly, in the control group with normal nutritional status parameters, significant negative relationship between 25(OH)D and chemerin was found. In overweight and obese children we observed a tendency for negative association between chemerin and 25(OH)D levels, but only after improvement in vitamin D status after six months of supplementation.

The relationships between excess body fat, insulin resistance, inflammation, dyslipidaemia and vitamin D deficit are intensively studied. Obesity-related insulin resistance and low-grade chronic inflammation seem to be the most important predictive factors for the development of complications of overweight and obesity such as metabolic syndrome, type 2 diabetes mellitus, cardiovascular disease and osteopenia/osteoporosis (47). The associations between vitamin D status and insulin sensitivity parameters in obese pediatric population have been previously reported (12, 48–53). The effects of vitamin D on glucose homeostasis, exerted mainly by its active form 1,25-dihydroxycholecalciferol, include the increase of peripheral and hepatic glucose uptake, the improvement in synthesis and secretion of insulin, the protection of β-cells from cytokine induced apoptosis and attenuation of inflammation (47, 54–56). It has been confirmed that insulin resistance affects obesity-related pro-atherogenic changes in lipid profile (57, 58). Insulin resistance impairs the function of insulin-dependent hormone-sensitive lipase and lipoprotein lipase, which are involved in lipid metabolism, whereas insulin cannot act effectively due to obesity and dyslipidaemia (59, 60). The study by Wang et al. (61) including nearly three hundred prepubertal and pubertal, normal and overweight/obese children and adolescents, demonstrated significant association between HOMA-IR and BMI and serum 25(OH)D level based on the stepwise multiple linear regression analysis of age, sex, pubertal maturation, BMI, WHtR, TG, total cholesterol, HDL-C, LDL-C, 25(OH)D and HOMA-IR. Several studies showed also the association between lipid profile, CRP, uric acid and vitamin D deficit, but the serum 25(OH)D level, predictive for vitamin D deficit-dependent metabolic disorders, has not been strictly defined (11, 62–68). Reis et al. (69), based on cross-sectional analysis of a group of more than three thousand adolescents, reported that individuals with 25(OH)D levels lower than 15 ng/ml are more likely to have fasting hyperglycemia, low HDL-C, hypertriglyceridemia, hypertension and metabolic syndrome compared to adolescents with 25(OH)D levels above 26 ng/ml. Rusconi et al. (62), among a group of more than one hundred obese children with 25(OH)D levels above or below 20 ng/ml, found higher total cholesterol and LDL-C levels in the group with vitamin D deficit. It should be noted that the stage of puberty also affects the relationship between vitamin D and the components of metabolic syndrome in obese children (53, 70, 71). Pires et al. (53) reported that the significant increase in TG, fasting insulin and HOMA-IR, observed during puberty, was related to a decrease in 25(OH)D levels independent of sex, body mass and pubertal Tanner stage.

Metabolic effects of vitamin D supplementation seem to be dependent on the dose and time of intervention. Our analysis showed that six months of vitamin D supplementation between 2000 to 4000 IU/day led mainly to a significant decrease in CRP, total cholesterol and aminotransferases activity. We found also that the increase in 25(OH)D levels resulted in a decrease in fasting glucose and insulin secretion at 120’ during the OGTT. Moreover, we noticed that after improvement of 25(OH)D level, a tendency for negative correlation between vitamin D status and chemerin appeared. Nader et al. (11) showed that twelve weeks of 2000 IU/day vitamin D supplementation in obese adolescents did not lead to detectable changes in fasting glucose, fasting insulin, HOMA-IR, lipids and highly sensitive CRP. On the other hand, the retrospective observational study by Pecoraro et al. (9), including overweight and obese children with 25(OH)D levels below 25 ng/ml, who underwent oral vitamin D supplementation (100 000 IU, one vial/month) for six months, indicated that vitamin D supplementation was associated with a significant decrease in total cholesterol, LDL-C and ALT serum levels and an increase in HDL-C.

In our study we also aimed to investigate the role of chemerin as a factor which could link vitamin D, insulin resistance and dyslipidaemia in overweight and obese children and adolescents. Those dependencies are not widely described, but some experimental studies suggest that protective effects of vitamin D supplementation could be exerted by reduction in chemerin levels (13, 14, 72). The number of clinical studies in that field is scarce. The cross-sectional study by Reyman et al. (12) revealed that vitamin D deficient obese children have significantly lower insulin sensitivity coexisted with higher chemerin, cathepsin S and soluble vascular adhesion molecule, which are known as pro-inflammatory, pro-diabetic and pro-atherogenic factors.

The mechanisms explaining the relationship between vitamin D status and chemerin are still not fully understood. Our multivariable backward linear regression models revealed WHtR and ALT activity as factors significantly positively associated with chemerin levels, both baseline and after six months of vitamin D supplementation. Based on our results we could presume that the relation between chemerin and body fat and hepatic steatosis is stronger than its association with vitamin D, especially in vitamin D deficient obese children (median 25(OH)D 16 ng/ml). Improvement of vitamin D status in our study group (median 25(OH)D 27.1 ng/ml) revealed a tendency for negative relationship between 25(OH)D and chemerin. In our control group with normal body weight and median 25(OH)D 25.7 ng/ml the significant negative correlation between 25(OH)D and chemerin levels was observed.

The study by Niklowitz et al. (25), including nearly one hundred obese children, confirmed that weight loss was associated with a decrease of chemerin levels and improvement of metabolic syndrome parameters such as insulin, HDL-C and TG. The associations between chemerin and weight loss were reported in several studies in obese pediatric and adult population (73–77). A study by Liu et al. (75) conducted among obese female adolescents showed that chemerin reduction achieved as a result of lifestyle changes correlated positively with fasting glucose, fasting insulin, HOMA-IR, TG and total cholesterol.

Gad et al. (78) revealed higher chemerin serum levels in obese children with metabolic syndrome. In his study chemerin positively correlated with fasting blood glucose and negatively with HDL-C. Other studies confirmed positive relationship between chemerin and insulin and HOMA-IR (25–27). In our study chemerin was related to insulin levels secreted in the response to OGTT, this relationship was present both before and after six months of vitamin D supplementation. Our study showed also positive correlation between chemerin and TG and TG/HDL-C ratio after the intervention.

Our study revealed positive correlation between chemerin levels and ALT activity. Elevated liver enzymes are the markers of non-alcoholic fatty liver disease (NAFLD), which is considered a liver manifestation of metabolic syndrome (79). Hamza et al. (44) among a group of fifty obese children also found a positive correlation between chemerin and liver enzymes, moreover he noticed that the increase in chemerin levels correlated positively with NAFLD severity.

Our study showed that vitamin D and chemerin are involved in metabolic processes. Taking into account that both of them affect adipose tissue and bone tissue, there may be some considerable overlap between their action, but it requires further investigation. We intend to continue our study on larger population and in longer follow-up.

## Conclusion

Our study confirmed that vitamin D has positive effect on metabolic profile in overweight and obese children. The relationship between vitamin D and chemerin is not clear, nevertheless we have observed a tendency to decrease chemerin concentrations after improving vitamin D status, even without a significant reduction in body fat mass. Taking into account the role of chemerin as early indicator of obesity-related diseases, the studies in this field seem to be valuable.

## Data availability statement

The original contributions presented in the study are included in the article/supplementary material. Further inquiries can be directed to the corresponding author.

## Ethics statement

The studies involving human participants were reviewed and approved by Bioethics Committee at the Medical University of Warsaw (decision number KB/257/2013). Written informed consent to participate in this study was provided by the participants’ legal guardian/next of kin.

## Author contributions

MK, EW-S and MR contributed to conception and design of the study. MK and MR organized the database. MK and EW-S prepared the tables. AM performed anthropometric measurements. MK and AS-E took measurements of serum chemerin levels. MK, EW-S and MS performed statistical analysis. MK and EW-S wrote the first draft of the manuscript. BP, AK supervised the work. MK and EW-S wrote the final version of the manuscript. All authors contributed to the article and approved the submitted version.
